# Harnessing the patient voice in prostate cancer research: Systematic review on the use of patient‐reported outcomes in randomized controlled trials to support clinical decision‐making

**DOI:** 10.1002/cam4.3018

**Published:** 2020-04-25

**Authors:** Mieke Van Hemelrijck, Francesco Sparano, Lisa Moris, Katharina Beyer, Francesco Cottone, Mirjam Sprangers, Fabio Efficace

**Affiliations:** ^1^ School of Cancer and Pharmaceutical Sciences Translational Oncology and Urology Research (TOUR) King’s College London London UK; ^2^ Data Center and Health Outcomes Research Unit, Italian Group for Adult Hematologic Disease (GIMEMA) Rome Italy; ^3^ University Hospitals Leuven Leuven Belgium; ^4^ Department of Medical Psychology Amsterdam University Medical Centers Location Academic Medical Center University of Amsterdam Amsterdam Public Health Research Institute Amsterdam The Netherlands

**Keywords:** outcome measurement, PROs, prostate cancer, quality of life, RCT

## Abstract

**Background:**

Given the growing importance of patient‐reported outcomes (PROs) as part of “big data” in improving patient care, there is a need to provide a state‐of‐the‐art picture of the added value of using PROs in prostate cancer (PCa) randomized controlled trials (RCTs). We aimed to synthetize the most recent high‐quality PRO evidence‐based knowledge from PCa RCTs and to examine whether quality of PRO reporting in PCa research improved over time.

**Methods:**

We conducted a systematic literature search using PubMed, from April 2012 until February 2019. For benchmarking purposes, we also included RCTs identified in our previously published review of RCTs (2004‐2012). Methodology for study identification and evaluation followed standardized criteria and a predefined data extraction form was used to abstract information. PRO quality of the studies was evaluated using the International Society of Quality of Life Research (ISOQOL) recommended criteria.

**Results:**

A total of 55 new RCTs were published between April 2012 and February 2019. About 24 (43.6%) RCTs were found to be of high‐quality regarding PRO assessments. Of these, 13 (54.2%) have been reported in the most recent European Association of Urology (EAU) PCa Guidelines. Overall QoL and sexual, urinary, and bowel function were the most commonly reported PROs. FACT‐P, EPIC‐26, and EORTC QLQ‐C30 and/or its module PR25 were most frequently used as measurement tools. An overall improvement in the completeness of PRO reporting was noted over time.

**Conclusion:**

Many PRO trials are currently not included in the EAU guidelines. Our findings suggest that there has to be a better consensus on the use of PRO data for PCa patients, which will then be reflected in the PCa Guidelines and future data collection. Homogeneity in PROs collection and measurement tools will in turn enable “big data” Consortia to increase the patients’ voice in clinical research.

## Graphical abstract

Patient reported outcomes need to become the standard in prostate cancer RCTs. There is need for consensus on the use of PRO data, which will reflect their use in the guidelines and future data collection.

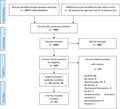

## 1 INTRODUCTION

Receiving a diagnosis of cancer and undergoing its subsequent treatments does not only have a detrimental impact on physical function, but may also affect psychological and social well‐being of the individual. Patient‐reported outcomes (PROs) addressing these domains of well‐being (including both physical and psychosocial components) are increasingly being incorporated in randomized controlled trials (RCTs) to assess the effectiveness of cancer treatments. A recent Cochrane systematic review on psychosocial well‐being and care needs of people with cancer ^1^ concluded that there is a need not only for more uniformity in outcomes and reporting, but importantly also for combining PROs with objective clinical outcomes. These conclusions are also in line with a recent report of the US Food and Drug Administration and the Critical Path Institute—as they are “committed to collaborate with international drug development stakeholders to identify rigorous methods to incorporate the patient perspective into the development of cancer therapeutics.”^2^

In the context of prostate cancer (PCa), we have previously evaluated the completeness of PRO reporting of 65 RCTs published between January 2004 and March 2012, which reported on PROs.^3^ Significant improvements in PRO quality reporting over time were observed and it was estimated that about 20% of PRO RCTs had provided solid PRO data to allow health policy makers and clinicians to make a critical appraisal. Since 2012, over 800 new studies have been published on PCa and PROs, according to PubMed. Since then, PIONEER, an IMI2 funded pan‐European public private partnership led by the European Association of Urology (EAU), is in the process of defining core outcome sets for PCa in the context of the patient's treatment pathway—whereby PROs from RCTs as well as real world evidence will be included. Core outcome sets for PCa will provide homogeneity in clinician reported outcomes and patient‐reported outcomes measures (PROMs) (in terms of outcomes and their definitions) to help Guidelines Offices with summarizing study findings and generate evidence‐based recommendations for the PCa treatment pathway. PIONEER’s goal is to ensure the optimal care for all European men diagnosed with PCa by unlocking the potential of “big data” and “big data analytics.”^4^, ^5^

Given the increasing importance of the use of PROs as part of “big data” in improving patient care,^6^ there is a need to provide evidence‐based PRO information that may be used to facilitate clinical decision‐making. The main objective of this study was to synthetize most recent high‐quality PRO data from PCa RCTs, that is, those studies most likely to robustly inform patient care through for example inclusion in the EAU PCa guidelines. The latter is a useful indication of quality of clinical studies with an impact on the PCa patient experience as these urological clinical guidelines are used worldwide to disseminate recommended PCa treatment pathways. The EAU PCa guidelines are updated on an annual basis using a broad and comprehensive literature search and are based on modified version of the Oxford Center for Evidence‐based Medicine Levels of Evidence. Any flaws in the evidence used to support any given recommendation are taken into account and hence reflect on the quality of PRO reported information. Details of the search methodology can be found here.^7^ A secondary objective of the current study was to assess whether completeness of PRO reporting in PCa research improved over time.

## 2 MATERIALS AND METHODS

### 2.1 Search strategy and identification of studies

We conducted a systematic literature search using PubMed, from April 2012 until February 2019. Methodology for study identification and evaluation followed standardized criteria used in the Patient‐Reported Outcomes Measurements Over Time In Oncology (PROMOTION) Registry (http://promotion.gimema.it). Study abstracts and additional records identified through hand searching of the literature were screened by two independent reviewers, following study selection criteria (see further for additional details). Then, full‐text articles selected were assessed for eligibility. This was previously described in the systematic review on PROs in PCa RCTs covering the years 2004‐April, 2012.^3^ For the purpose of the current review, we used the same search terms as in our previous work^3^: *("quality of life" OR “health related quality of life” OR "health status" OR “health outcomes” OR “patient outcomes” OR “depression” OR “anxiety” OR “emotional” OR “social” OR “psychosocial” OR “psychological” OR “distress” OR “social functioning” OR “social wellbeing” OR “emotional” OR “patient reported symptom” OR "patient reported outcomes" OR pain OR fatigue OR “patient reported outcome” OR "PRO" OR "PROs" OR "HRQL" OR "QOL" OR "HRQOL" OR “symptom distress” OR “symptom burden” OR “symptom assessment” OR “functional status” OR sexual OR functioning) AND prostate*. The search strategy was restricted to RCTs. In case of multiple publications from the same RCT, all relevant data possibly published in secondary articles were combined.

### 2.2 Selection criteria

Only English‐language reports of RCTs comparing conventional treatments and involving adult men with PCa were included—irrespective of disease stage. The minimum, overall sample size (combined treatment arms) was set at 50 patients. Screening studies or those involving patients with benign disease were excluded. We did not consider conference abstracts as these typically report insufficient information on PRO methodology and outcomes. RCTs of interventions that were psychological, behavioral, complementary, or alternative were also excluded.

We included all studies evaluating a PRO either as a primary or secondary endpoint—either as a multidimensional QoL outcome or a single dimensional outcome, such as symptoms. Those studies evaluating only treatment adherence or satisfaction were excluded. Details on search strategy and selection process were documented according to the PRISMA guidelines.^8^

### 2.3 Methods of evaluation of studies

Three reviewers (FS, LM, and KB) extracted information from the identified studies. Each study was evaluated independently by two of these reviewers. All data were entered by the reviewers into a password protected online database (REDCap)^9^ by completing a predefined electronic‐data extraction form (eDEF). Full details on information contained in the PROMOTION eDEF are reported in the appendix. A double‐blind data entry procedure was performed as each reviewer completed the eDEF independently. Discrepancies in evaluations were electronically recorded and when disagreements occurred in the evaluation of any item included in the eDEF, the reviewers revisited the paper to reconcile any differences. If no consensus was achieved, a fourth reviewer (FE) was consulted. For every included RCTs, their inclusion in the EAU PCa guidelines was checked by manual searches of the references and corresponding sections.

### 2.4 Type of data extraction and data analysis

For the purpose of this review, the following types of information were considered: (a) basic trial characteristics, (b) clinical and PRO characteristics, and (c) elements of PRO reporting based on recommendations from the International Society of Quality of Life Research (ISOQOL)^10^ and the CONSORT‐PRO extension.^11^ Quality of PRO reporting was evaluated with the ISOQOL checklist, which comprises a common set of 17 key issues regardless of PRO being a primary or secondary endpoint. Eleven additional issues were considered when a PRO was a primary endpoint of the study. Each item of the ISOQOL checklist was rated as “yes” if documented in the publication (scored as 1) or “no” if not documented (scored 0). To further refine the investigation of the accuracy of reporting, we divided the ISOQOL item addressing the problem of missing data into two (ie reporting the extent of missing data and reporting statistical approaches for dealing with missing data). We thus rated each RCT with a score ranging from 0 to a maximum of 18 (RCT with PRO as a secondary endpoint) or 29 (PRO as primary endpoint); in both cases, the higher the score the better the quality of the PRO reporting. Identification of high‐quality PRO studies was based on previously defined criteria.^3^ Specifically, we defined as high‐quality PRO studies those which, at the same time, satisfied at least two‐thirds of the recommended criteria (12 for RCT with PRO as secondary endpoint and 20 for PRO as primary endpoint) and addressed three mandatory issues: study patients characteristics and baseline PRO scores described, documentation of PRO instrument validity, and missing data reported. In addition, we checked whether those studies considered as high‐quality RCTs were included in the most recent EAU PCa Guidelines.^12^, ^13^

Main characteristics of eligible studies (eg disease stage, type of PRO endpoint) were reported by proportions and means, according to the type of variable. Differences between studies were assessed using the chi‐square test. Based on the ISOQOL checklist score, comparisons between RCTs selected for this review and those included in a previous study^3^ were performed to examine whether the completeness of PRO reporting in PCa trials has improved over time. We also compared the overall level of PRO reporting according to the CONSORT‐PRO extension, between the RCTs published before and after the publication of this guideline. All tests were two‐sided and statistical significance was set at α = 0.05. Analyses were performed by SAS software v. 9.4 (SAS Institute Inc).

## 3 RESULTS

**FIGURE 1:**
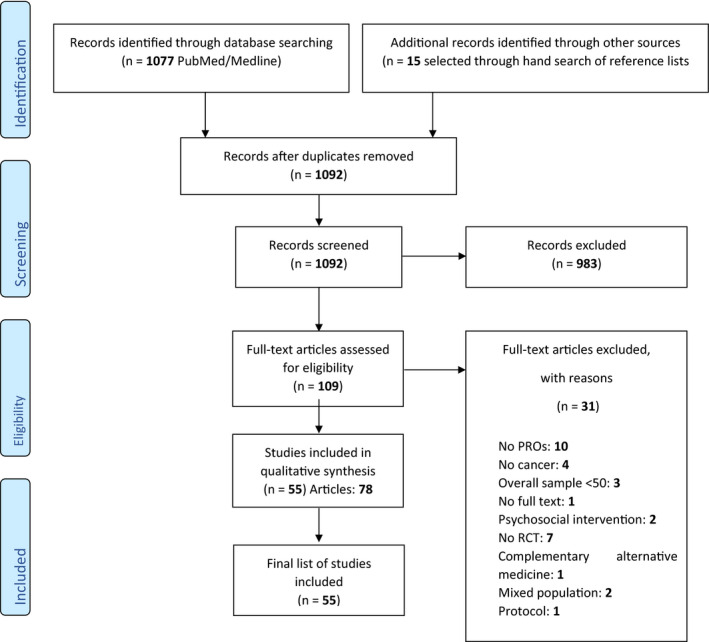
PRISMA flow chart of literature search results of Prostate Cancer Randomised Controlled Trials. PRO, patient‐reported outcomes

A total of 55 new RCTs were published between April 2012 and February 2019 (Figure 1), of which the majority were international trials (ie more than one country) (60%). An overview of trial characteristics, as compared to the data from January 2004 to March 2012 is shown in Table 1. The duration of PRO assessment has increased, with 63.6% of trials reporting a period of more than 1 year as compared to 43.1% of trials previously (*P* < .006). Most RCTs reported on PROs as a secondary endpoint (70.9% vs 60% previously; *P* = .212). However, the prevalence of RCTs with a sample size ≥200 has increased over time, with 69.1% of RCTs including more than 200 men with PCa, as compared to 52.3% previously (*P* = .062).

**TABLE 1:** Overview of RCT characteristics as compared to our previous systematic review^a^

Variable	RCTs January 2004‐March 2012 (n=65)	RCTs April 2012‐February 2019 (n=55)	Total n (%)	*P* value (two sided)
International (if more than one country)				
No	46 (70.8)	22 (40)	68 (56.7)	<.001
Yes	19 (29.2)	33 (60)	52 (43.3)	
Industry supported (fully or in part)				
No	31 (47.7)	16 (29.1)	47 (39.2)	.038
Yes	34 (52.3)	39 (70.9)	73 (60.8)	
Disease stage				
Only metastatic/advanced	21 (32.3)	27 (49.1)	48 (40)	.160
Only nonmetastatic/local	31 (47.7)	21 (38.2)	52 (43.3)	
Both	13 (20)	7 (12.7)	20 (16.7)	
Secondary paper on PRO				
No	47 (72.3)	37 (67.3)	84 (70)	.549
Yes	18 (27.7)	18 (32.7)	36 (30)	
Length of PRO assessment during RCT				
Up to 6 mo	22 (33.8)	10 (18.2)	32 (26.7)	.006
Up to 1 y	15 (23.1)	6 (10.9)	21 (17.5)	
More than 1 y	28 (43.1)	35 (63.6)	63 (52.5)	
Unknown	0 (0)	4 (7.3)	4 (3.3)	
Overall study sample size				
≤200	31 (47.7)	17 (30.9)	48 (40)	.062
>200	34 (52.3)	38 (69.1)	72 (60)	
Type of treatment used^b^				
Radiotherapy	21 (32.3)	17 (30.9)	38 (31.7)	
Surgery	10 (15.4)	4 (7.3)	14 (11.7)	
Chemotherapy	13 (20)	10 (18.2)	23 (19.2)	
Targeted therapy	0 (0)	12 (21.8)	12 (10)	
Hormonal therapy	25 (38.5)	24 (43.6)	49 (40.8)	
Other	22 (33.9)	4 (7.3)	26 (21.7)	
PRO endpoint				
Primary	26 (40)	16 (29.1)	42 (35)	.212
Secondary	39 (60)	39 (70.9)	78 (65)	
Most frequently used PRO instruments^b^				
EORTC Questionnaires	18 (27.7)	16 (29.1)	34 (28.3)	
FACT Questionnaires	13 (20)	21 (38.2)	34 (28.3)	
BPI	5 (7.7)	13 (23.6)	18 (15)	
VAS or LASA Questionnaires	8 (12.3)	3 (5.5)	11 (9.2)	
IPSS	2 (3.1)	7 (12.7)	9 (7.5)	
SF‐36 Questionnaires	2 (3.1)	6 (10.9)	8 (6.7)	
EPIC	0 (0)	7 (12.7)	7 (5.8)	
EQ‐5D	1 (1.5)	5 (9.1)	6 (5)	

Abbreviations: BPI, Brief Pain Inventory; EORTC, European Organization for Research and Treatment of cancer; EPIC, Expanded Prostate Cancer Index Composite; EQ‐5D, EuroQol‐5 Dimensions; FACT, Functional Assessment of Cancer Therapy; LASA, Linear Analog Scale Assessment; PRO, Patient‐Reported Outcomes; RCT, Randomized Controlled Trial; VAS, Visual Analog Scale.

^a^ Efficace et al *Eur Urol*. 2014;66(3):416‐427.

^b^ More than one category can be chosen.

To assess the clinical impact of the RCTs, we first identified the proportion of high‐quality RCTs with respect to PRO reporting, which was 43.6%, an increase since 2012 when it was 20% (*P* < .01). Table 2 provides more information about these high‐quality RCTs (n = 47 reports—describing study results of 24 different RCTs). The majority of trials (15/24) were conducted in the advanced PCa setting (ie locally advanced PCa, metastatic PCa, metastatic castration‐resistant and nonmetastatic castration‐resistant PCa). Some RCTs included patients with different PCa stages, so that it was not possible to categorize these RCTs according to localized, locally advanced, or metastatic PCa. Six studies focussed exclusively on men with localized PCa. In these high‐quality RCTs, PROs encompassed Health‐Related Quality of Life (HRQoL) or symptoms such as erectile, bladder, or bowel function. The most frequently used measures were Functional Assessment of Cancer Therapy‐Prostate (FACT‐P) (9/24), EORTC QLQ‐C30 and/or its module QLQ‐PR25 (5/24), and Expanded Prostate Cancer Index Composite (EPIC) (5/24).

**TABLE 2:** Patient‐Reported Outcomes (PROs) and PRO measurements (PROMs) in the high‐quality PRO RCTs for prostate cancer

Name of study	First author	PCa risk category	PRO	PROM
PREVAIL, 2015	Loriot ^27^	mCRPC	HRQoL Pain	FACT‐P EQ‐5D Brief Pain Inventory Short
PREVAIL, 2014^a^	Beer^28^	mCRPC	QoL	FACT‐P
PREVAIL, 2017	Devlin^29^	mCRPC	HRQoL	EQ‐5D
REACTT, 2015	Patel^30^	Nonmetastatic PCa (GS ≤ 7, PSA < 10)	Erectile function Patients’ treatment satisfaction Self‐esteem (baseline) Prostate‐specific QoL	IIEF‐EF EDITS questionnaire SEAR questionnaire EPIC‐26
REACTT, 2013	Montorsi^31^	Nonmetastatic PCa (GS ≤ 7, PSA < 10)	Erectile Function Sexual encounter	IIEF‐EF score SEP‐1/ SEP‐2 questions
RTOG‐0126, 2015	Bruner^32^	cT1b‐2b, (+GS2‐6, PSA10‐20 OR GS7, PSA < 15)	Bladder function Bowel function Erectile function	FACE questionnaire FACE questionnaire IIEF questionnaire
RTOG‐0126, 2013	Michalski^33^	cT1b‐2b, (+GS2‐6, PSA10‐20 OR GS7, PSA < 15)	Acute GI/GU toxicity Late GI/GU toxicity	RTOG/EORTC late morbidity scoring system
RTOG‐0126, 2018^a^	Michalski^34^	cT1b‐2b, (+GS2‐6, PSA10‐20 OR GS7, PSA < 15)	/	/
AFFIRM, 2014	Fizazi^35^	mCRCP	Pain HRQoL improvement HRQoL deterioration	BPI‐SF FACT‐P FACT‐P
AFFIRM, 2015	Cella^36^	mCRPC	HRQoL	FACT‐P
AFFIRM, 2012^a^	Scher^37^	mCRPC	QoL	FACT‐P
AFFIRM, 2017	Armstrong^38^	mCRPC	/	/
COU‐AA‐302 phase 3, 2013	Basch^39^	mCRPC	Pain HRQoL	BPI‐SF questionnaire FACT‐P questionnaire
COU‐AA‐302 phase 3, 2012^a^	Ryan^40^	mCRPC	HRQoL Pain	FACT‐P BPI‐SF
PROTECT, 2013	Beer^41^	Nonmetastatic androgen dependent PCa with PSA relapse and GS ≥ 7 after RP	QoL	BFI score LASA score, GRoC scale, symptoms checklist
PROTECT, 2011	Beer^42^	Nonmetastatic androgen dependent PCa with PSA relapse and GS ≥ 7 after RP	/	/
SWOG‐9346, 2013^a^	Hussain^43^	mCRPC	HRQoL	QoL questionnaire (SWOG)
NR	Mason^44^	T2b‐T4N0M0, GS ≥ 7, or PSA ≥ 10	LUTS relief QoL (urinary symptoms)	IPSS
TROG 03.04 RADAR, 2012	Denham^18^	cT2b–4N0M0 or T2a + GS≥7 + PSA≥10	QoL	EORTC QLQ‐C30 + EORTC QLQ‐PR‐25 questionnaires
TROG 03.04 RADAR, 2014	Denham^45^	cT2b–4N0M0 or T2a + GS≥7 + PSA≥10	/	/
TROG 03.04 RADAR, 2012	Denham^46^	cT2b–4N0M0 or T2a + GS≥7 + PSA≥10	Dysfunctional rectal symptoms	EORTC QLQ‐PR25 questionnaire
NCT00884273, 2012	Axcrona^47^	PCa all stages	LUTS relief QoL improvement	IPSS
CHHiP, 2015^a^	Wilkins^17^	pT1b–T3aN0M0	Overall bowel bother Overall urinary bother Overall sexual bother General HRQoL	UCLA‐PCI, EPIC instrument FACT‐P, SF‐36, SF‐12
CHHiP, 2016^a^	Dearnaley^48^	pT1b–T3aN0M0	Patient reported outcome	UCLA‐PCI, EPIC instrument
ACTRN12611000661976^a^	Yaxley^15^	≤T2cN0M0	Urinary function Sexual function Pain Physical and mental functioning Fatigue Bowel function Cancer‐specific distress Psychological distress Time to return to work	EPIC score EPIC/ IIEF score Surgical pain scale SF‐36 Vitality domain SF‐36 EPIC score RIES scale HADS score /
NCT00866554	Gaudet^49^	T1c‐T2b, GS: 6 or 7(3 + 4), PSA ≤ 15	Acute and late effects on sexual function Urinary toxicity	IPSS + EPIC score IPSS + EPIC score
TROG 03.06 and VCOG PR 01‐03, 2017	Duchesne^50^	PSA relapse after treatment or group two of asymptomatic men unsuitable for curative treatment because of age, comorbidity, or locally advanced disease	Global HRQoL	EORTC QLQ‐C30, EORTC QLQ‐PR25
TROG 03.06 and VCOG PR 01‐03, 2016^a^	Dushesne^51^	PSA relapse after treatment or group two of asymptomatic men unsuitable for curative treatment because of age, comorbidity, or locally advanced disease	Global HRQoL	EORTC QLQ‐C30, EORTC QLQ‐PR25
MRC PATCH trial (PR09), 2016	Gilbert^52^	cT3‐4cN + M0 or TxNxM1	Global health status/QoL Urinary, bowel and sexual symptoms and function and hormone‐related symptoms	EORTC QLQ‐C30, QLQ‐PR25
MRC PATCH trial (PR09), 2013	Langley^53^	cT3‐4cN + M0 or TxNxM1	Adverse events (not patient reported)	Not applicable
MRC PATCH trial (PR09), 2016	Langley^54^	cT3‐4cN + M0 or TxNxM1	/	/
ASCENDE‐RT trial, 2016	Rodda^55^	High‐risk PCa: T3aN0M0, GS 8‐9, PSA > 20	HRQoL	SF36v2
ASCENDE‐RT trial, 2013^a^	Morris^56^	High‐risk PCa: T3aN0M0, GS 8‐9, PSA > 20	/	/
PROSPER, 2018^a^	Hussain^57^	nmCRPC	QoL assessments	/
PROSPER, 2019	Tombal^58^	nmCRPC	Pain progression HRQoL	BPI‐SF questionnaire EORTC QLQ‐PR25
RTOG 0938, 2018	Lukka^59^	Low risk PCa (cT1‐2aN0M0, PSA < 10, GS 2‐6)	Bowel and urinary PROs % of patients with >5 point reduction in the EPIC bowel domain at 1 y % of patients with >2 point reduction in EPIC urinary domain at 1 y Sexual and hormonal toxicity Acute/Late GI/GU toxicity	EPIC‐50 EPIC 50 EPIC 50 EPIC 50 EPIC 50
COMET‐2, 2018	Basch^60^	mCRPC	Rate of pain response	BPI reports
SPARTAN, 2018	Saad^16^	nmCRPC	HRQoL: PCa symptoms, pain‐related symptoms, and overall QoL HRQoL: mobility, self‐care, usual activities, pain, discomfort, and anxiety or depression	EQ‐5D‐3L FACT‐P
SPARTAN, 2018^a^	Smith^61^	nmCRPC	/	/
NCT 02135357, 2018	Khalaf^62^	mCRPC	Patient reported HRQoL Depression symptoms Cognitive function	FACT‐P PHQ‐9 MoCA
NCT 02135357, 2018	Annala^63^	mCRPC	/	/
SWOG S0421, 2018	Unger^64^	mCRPC	Palliation of worst pain Improvement of functional status Vitality General QoL Analgesic use	BPI inventory FACT‐P TOI SF‐36 Energy/vitality scale EORTC QLQ‐C30 (Pain medication logs)
SWOG S0421, 2013	Quinn^65^	mCRPC	/	/
CHAARTED, 2015^a^	Sweeney^66^	Metastatic hormone‐sensitive PCa	/	/
CHAARTED, 2018	Morgans^67^	Metastatic hormone‐sensitive PCa	Overall QoL Treatment and disease‐related QoL Adverse effect of taxanes treatment Fatigue Pain	FACT‐P FACT‐Taxane FACIT‐Fatigue BPI
LATITUDE, 2017^a^	Fizazi^68^	Metastatic hormone‐sensitive PCa	Time to pain progression	BPI‐SF
LATITUDE, 2018	Chi^69^	Metastatic hormone‐sensitive PCa	Pain Fatigue Disease‐related QoL HRQoL	BPI‐SF BFI FACT‐P EQ‐5D‐5L

Abbreviations: BPI, Brief Pain Inventory; EDITS, Erectile Dysfunction Inventory of Treatment Satisfaction; EORTC QLQ‐C30, European Organization for Research and Treatment of Cancer Quality of life questionnaire‐Core 30; EORTC QLQ‐PR25, European Organization for Research and Treatment of Cancer Quality of life questionnaire‐Prostate 25; EPIC, Expanded Prostate Index Composite questionnaire; EQ‐5D, EuroQol 5‐dimension; EQ‐5D‐3L, EuroQol 5‐dimension, 3‐level questionnaire; FACE, Functional Alterations due to Changes in Elimination; FACT‐P, Functional assessment of Cancer Therapy‐Prostate Cancer; HADS, Hospital Anxiety and Depression Scale; IIEF‐EF, International Index of Erectile Function‐Erectile Function; IPSS, International Prostate Symptom Score; MoCA, Montreal Cognitive Assessment;PHQ‐9, Patient Health Questionnaire‐9; RIES, Revised Impact of Events; SEAR, Self‐Esteem and Relationship questionnaire; SF‐36, Short‐Form 36; UCLA‐PCI, UCLA Prostate Cancer Index.

^a^ Included in the EAU prostate cancer guidelines.

To further assess the clinical impact of these RCTs, Table 3 reports on the influence of the reported HRQoL on the final treatment recommendation (as defined by the authors of each published RCT). As most RCTs reported clinical and HRQoL specific outcomes separately, Table 3 aims to combine the results as one final recommendation for each RCT. In the metastatic PCa group, new treatment options (ie second‐generation antiandrogens like enzalutamide and abiraterone) and the use of docetaxel resulted in significant improvements in both clinical (overall, progression‐free, and metastatic‐free survival) and HRQoL outcomes.

**TABLE 3:** Evaluation of HRQoL outcomes/recommendations for included high‐quality PROs

Name of trial	Treatment	HRQoL outcome	Clinical outcome	Treatment recommendations
PREVAIL* Loriot^27^ Beer^28^ Devlin^29^	Enzalutamide (160 mg/d) vs placebo	Enza: reduced risk of and delayed time to HRQoL deterioration, pain progression, and occurrence of SREs.		Enza is recommended in asymptomatic and minimally symptomatic, chemo‐naïve patients with mCRPC due to its positive effects on survival and HRQoL benefits.
			Significant delay in radiographic disease progression or death and need for cytotoxic chemotherapy	
		Significant benefits in terms of Pain/Discomfort and Anxiety/Depression (EQ‐5D)		
REACTT Patel^30^ Montorsi^31^	9 mo tadalafil 5 mg once daily vs tadalafil 20 mg on demand vs placebo	Early chronic dosing after nsRP increases and accelerates EF recovery and improves patients’ QoL.		Tadalafil treatment may contribute to the recovery of EF after RP
		Improvements in IIEF‐EF and successful intercourse during 9 mo tadalafil once daily were not sustained 6w after drug cessation.	Protection from penile length loss	
RTOG‐0126* Bruner^32^ Michalski^33^ Michalski^34^	3D‐CRT vs IMRT	No difference in patient‐reported bowel, bladder, or sexual functions		The decision to deliver high radiation dose must be balanced against the risk of morbidity in the individual patient.
		IMRT: Lower incidence of acute GI or GU toxicity and a lower cumulative incidence of late grade 2b rectal toxicity		
		Increase in late grade 2 or greater GI and GU toxic effects.	Improvement in biochemical failure and distant metastases, but no improvement in OS.	
AFFIRM* Fizazi^35^ Cella^36^ Scher^37^ Armstrong^38^	Enzalutamide (160 mg/d) vs placebo	Reduction of the risk of SREs. Reduction of pain and increase in time to HRQoL deterioration		Enza improves both OS and well‐being and everyday functioning of patients with mCRPC (postchemo)
		Stabilization of patient HRQoL		
			Prolonged OS	
			PSA declines of any, ≥30%, and ≥50% within 90 d of Enza were strongly associated with the clinical benefit	
COU‐AA‐302 phase 3* Basch^39^ Ryan^40^	Abiraterone acetate + prednisone vs prednisone alone	Delay in patient‐reported pain progression and HRQoL deterioration		Abi + prednisone can be recommended for patients with mCRPC (prechemo)
			Improvement of radiographic PFS, a trend toward improvement of OS, and significant delay in clinical decline and initiation of chemotherapy	
PROTECT Beer^41^ Beer^42^	Sipuleucel‐T vs placebo	No clinically significant negative impact on QoL		Long‐term FU is needed to determine the effect on clinically important events
			No difference in biochemical failure	
SWOG‐9346, 2013* Hussain^43^	Intermittent vs continuous ADT	Intermittent therapy was associated with improved EF and mental health at 3 mo but not thereafter.	Too few events occurred to rule out significant inferiority of intermittent therapy	In this noninferiority trial, findings were statistically inconclusive
NR Mason^44^	Degarelix + RT vs Goserelin with bicalutamide + RT	Degarelix had more pronounced effects on LUTS in symptomatic patients	Noninferior efficacy of degarelix in terms of prostate shrinkage	Degarelix provides an alternative treatment for PCa patients who need neoadjuvant ADT before RT, especially for those having LUTS problems.
TROG 03.04 RADAR Denham^18^ Denham^45^ Denham^46^	STAS (6 mo) vs STAS + ZA vs ITAS (12 mo) vs ITAS + ZA	ITAS + RT causes adverse effects on some PROs but not on global QoL scores. Only hormone treatment‐related symptoms persisted at marginally higher frequencies. HDR‐BT boost adversely affected emotional function and financial problems.		Further follow‐up of the RADAR trial is needed before we can take our findings to the clinic
			No difference in PCa‐specific mortality between the 4 groups	
		ADT, ZA and increasing EBRT dose did not increase rectal or urinary dysfunction. The use of HDR‐BT increased urinary dysfunction.		
NCT00884273 Axcrona^47^	12 wk of degarelix (240/80 mg) vs goserelin (3.6 mg) + 28 d of bicalutamide.	Degarelix showed superiority in LUTS relief in symptomatic patients	Same reduction in total prostate volume	Degarelix can be considered as a useful approach to combined GnRH agonist plus antiandrogen for PCa patients in need of short‐term neoadjuvant ADT.
CHHiP* Wilkins^17^ Dearnaley^48^	Hypofractionated RT vs conventional RT	PROs were not significantly different between treatment groups for any of the endpoints		Hypofractionated RT using 60 Gy in 20 fractions is recommended as new standard of care for EBRT of localized PCa.
			Hypofractionated schedule is noninferior to the conventionally fractionated schedule for time to biochemical or clinical failure	
ACTRN12611000661976* Yaxley^15^	RARP vs RRP	No difference in domain‐specific QoL outcomes at 12 wk	No difference in pathological outcomes at 12 wk	Long‐term follow‐up is needed
NCT00866554 Gaudet^49^	Dutasteride 0.5 mg + Bicalutamide 50 mg + Tamoxifen 10 mg daily vs LHRH agonist + Bicalutamide daily	Less sexual toxicity compared to LHRH agonists prior to BT and for the first 6 mo after BT	Noninferior efficacy to LHRH agonist based regimens for prostate volume reduction prior to BT	D + B is therefore an option to be considered for prostate volume reduction prior to BT
TROG 03.06 and VCOG PR 01‐03* Duchesne^50^ G Dushesne ^51^	Immediate vs delayed ADT (PSA relapse only)	Early detriments in specific hormone treatment‐related symptoms with immediate ADT, but with no other demonstrable effect on overall functioning or HRQoL		Progression is delayed, but at a small cost in global QoL. The option can be discussed with men with a PSA relapse.
			Immediate receipt of ADT significantly improved OS	
MRC PATCH trial (PR09) Gilbert^52^ Langley^53^ RE Langley ^54^	Transdermal estradiol vs LHRH agonist	estradiol: better self‐reported QoL outcomes at 6 mo but increased gynecomastia		Provides further supporting evidence for the ongoing phase 3 trial
			Castrate testosterone concentrations similar to those achieved with LHRHa	
		Mitigating BMD loss	Castration levels of testosterone comparable with LHRHa administration	
ASCENDE‐RT trial* Rodda^55^ Morris^56^	LDR‐BT vs DE‐EBRT	LDR‐PB boost: more moderate to severe GU toxicity, urinary incontinence, and need for catheterization and a larger mean decline in HRQoL for physical and urinary function at 6 y.		Treatment should be individualized and requires careful consideration of the potential risks and benefits.
			LDR‐PB patients were twice as likely to be free of BF at a median follow‐up of 6.5 y	
PROSPER* Hussain^57^ Tombal^58^	Enzalutamide vs placebo		Significantly increased metastasis‐free survival	Enza is a treatment option that should be discussed in high‐risk, nmCRPC
		Benefit in delaying pain progression, symptom worsening, and decrease in functional status		
RTOG 0938, 2018 Lukka^59^	Two ultrahypofractionated RT schemes	Both schemes are well tolerated and bowel, urinary, and sexual PROs are comparable to those for standard RT		Longer follow‐up is required
COMET‐2, 2018 Basch^60^	Cabozantinib vs mitoxantrone‐prednisone	Cabozantinib treatment did not improve pain palliation		Enrollment was terminated
SPARTAN* Saad^16^ MR Smith ^61^	Apalutamide vs placebo	HRQoL was maintained after initiation of apalutamide treatment		Apalutamide provides clinical benefit in the treatment of men with nmCRPC
			Metastasis‐free survival and time to symptomatic progression were significantly longer	
NCT 02135357 Khalaf^62^ Annala^63^	Abiraterone vs enzalutamide	PROs favored Abi with differences in FACT‐P and PHQ‐9 scores. Differences in the total FACT‐P score only in the elderly subgroup.		Abi and Enza are standard first‐line treatment options for mCRPC with similar efficacy but different side‐effect profiles. Administration should be discussed with each patient individually.
			Enza: superior PSA responses but no differences in progression‐free survival	
CHAARTED* Sweeney^66^ Morgans^67^	ADT + Docetaxel vs ADT alone		Six cycles of docetaxel at the beginning of ADT resulted in significantly OS than ADT alone.	For patients with hormone‐sensitive metastatic PCa, who are fit enough ADT + docetaxel can be considered
		Both arms reported a similar minimally changed QoL over time, suggesting that ADT + Docetaxel is not associated with a greater long‐term negative impact on QoL		
LATITUDE* Fizazi^68^ Chi^69^	ADT + abiraterone acetate + prednisone vs ADT alone		Addition of abiraterone acetate increased OS and radiographic progression‐free survival	Treatment with ADT plus abiraterone acetate and prednisone could be considered a new option for standard of care for patients with metastatic castration‐naïve PCa
		Addition of abiraterone acetate improved overall PROs by consistently showing a clinical benefit in the progression of pain, PCa symptoms, fatigue, functional decline, and overall HRQoL.		
SWOG S0421 Unger^64^ Quinn^65^	Docetaxel + Atrasentan vs Docetaxel + placebo	No substantial treatment arm differences for pain and functional status		Docetaxel remains one of the standard options for CRPC. Endothelin inhibitors do not have an established role.
			Atrasentan did not improve PFS or OS	

To date, 14 of the 47 publications have been included in the EAU PCa Guidelines^12^, ^13^—reflecting 13 different RCTs (Table 2).

In terms of methodology, there was still a lack of information on mode of administration of the PRO tool and methods of data collection over time (83.6% vs 76.9%; *P* = .360). However, there was an improvement in the reporting of the evidence for PRO instrument validity and reliability (80% vs 66.1%; *P* = .007). More RCTs also identified PROs in the trial protocol and post hoc analyses (67.3% vs 20%; *P* < .001). In general, there was an improvement in terms of reporting methods and results for PROs in PCa RCTs. A detailed overview of the methodological assessment, as compared to our previous systematic review,^3^ is provided in Table 4.

**TABLE 4:** Level of patient‐reported outcomes (PRO) reporting as compared to our previous systematic review^3^

Variable	Category	RCTs January 2004‐March 2012 n = 65 (%)	RCTs April 2012‐February 2019 n = 55 (%)	Total n (%)	*P* value (two sided)
**Title and abstract**					
The PRO is identified as an outcome in the abstract	No	6 (9.2)	4 (7.3)	10 (8.3)	.699
	Yes	59 (90.8)	51 (92.7)	110 (91.7)	
(Additional standards only for PRO as primary outcome) The title of the paper is explicit as to the RCT including a PRO^a^	No	10 (38.5)	9 (56.3)	19 (45.2)	.252
	Yes	16 (61.5)	7 (43.8)	23 (54.8)	
**Introduction, background, and objectives**					
The PRO hypothesis is stated and should specify the relevant PRO domain if applicable	No	11 (16.9)	19 (34.5)	30 (25)	.082
	Yes	24 (36.9)	15 (27.3)	39 (32.5)	
	N/A (if explorative)	30 (46.2)	21 (38.2)	51 (42.5)	
(Additional standards only for PRO as primary outcome) The introduction contains a summary of PRO research that is relevant to the RCT^a^	No	3 (11.5)	7 (43.7)	10 (23.8)	.031
	Yes	23 (88.5)	9 (56.3)	32 (76.2)	
(Additional standards only for PRO as primary outcome) Additional details regarding the hypothesis are provided including the rationale for the selected domains, the expected directions of change, and the time points for assessment.^a^	No	22 (84.6)	12 (75)	34 (81)	.346
	Yes	4 (15.4)	4 (25)	8 (19)	
**Methods**					
*Outcomes*					
The mode of administration of the PRO tool and the methods of collecting data are described	No	50 (76.9)	46 (83.6)	96 (80)	.360
	Yes	15 (23.1)	9 (16.4)	24 (20)	
Electronic mode of PRO administration	No	15 (23.1)	5 (9.1)	20 (16.7)	.044
	Yes	0 (0)	2 (3.6)	2 (1.6)	
	N/A	50 (76.9)	48 (87.3)	98 (81.7)	
The rationale for choice of the PRO instrument used is provided	No	24 (36.9)	26 (47.3)	50 (41.7)	.252
	Yes	41 (63.1)	29 (52.7)	70 (58.3)	
Evidence of PRO instrument validity and reliability is provided or cited	No	22 (33.9)	11 (20)	33 (27.5)	.007
	Yes, for all PRO instruments	25 (38.4)	37 (67.3)	62 (51.7)	
	Yes, only for some PRO instruments	18 (27.7)	7 (12.7)	25 (20.8)	
The intended PRO data collection schedule is provided	No	6 (9.2)	5 (9.1)	11 (9.2)	.979
	Yes	59 (90.8)	50 (90.9)	109 (90.8)	
PROs are identified in the trial protocol; post hoc analyses are identified	No	52 (80)	18 (32.7)	70 (58.3)	<.001
	Yes	13 (20)	37 (67.3)	50 (41.7)	
The status of PRO as either a primary or secondary outcome is stated	No	9 (13.8)	3 (5.5)	12 (10)	.106
	Yes	48 (73.9)	49 (89)	97 (80.8)	
	Unclear	8 (12.3)	3 (5.5)	11 (9.2)	
(Additional standards only for PRO as primary outcome) A citation for the original development of the PRO instrument is provided^a^	No	11 (42.3)	3 (18.8)	14 (33.3)	.086
	Yes	7 (26.9)	10 (62.4)	17 (40.5)	
	Yes, only for some PRO instruments	8 (30.8)	3 (18.8)	11 (26.2)	
(Additional standards only for PRO as primary outcome) Windows for valid PRO responses are specified and justified as being appropriate for the clinical context^a^	No	7 (26.9)	14 (87.5)	21 (50)	<.001
	Yes	19 (73.1)	2 (12.5)	21 (50)	
*Sample size*					
(Additional standards only for PRO as primary outcome) There is a power sample size calculation relevant to the PRO based on a clinical rationale^a^	No	10 (38.5)	5 (31.2)	15 (35.7)	.412
	Yes	16 (61.5)	11 (68.8)	27 (64.3)	
*Statistical methods*					
There is evidence of appropriate statistical analysis and tests of statistical significance for each PRO hypothesis tested	No	2 (3.1)	3 (5.5)	5 (4.2)	.418
	Yes	22 (33.8)	13 (23.6)	35 (29.2)	
	N/A (If PRO hypotheses were not stated)	41 (63.1)	39 (70.9)	80 (66.6)	
The extent of missing data is stated	No	18 (27.7)	17 (30.9)	35 (29.2)	.699
	Yes	47 (72.3)	38 (69.1)	85 (70.8)	
Statistical approaches for dealing with missing data are explicitly stated	No	53 (81.5)	35 (63.6)	88 (73.3)	.027
	Yes	12 (18.5)	20 (36.4)	32 (26.7)	
(Additional standards only for PRO as primary outcome) The manner in which multiple comparisons have been addressed is provided^a^	No	19 (73.1)	11 (68.8)	30 (71.4)	.439
	Yes	7 (26.9)	5 (31.2)	12 (28.6)	
**Results**					
*Participant flow*					
A flow diagram or a description of the allocation of participants and those lost to follow‐up is provided for PROs specifically	No	41 (63.1)	24 (43.6)	65 (54.2)	.033
	Yes	24 (36.9)	31 (56.4)	55 (45.8)	
The reasons for missing data are explained	No	42 (64.6)	37 (67.3)	79 (65.8)	.760
	Yes	23 (35.4)	18 (32.7)	41 (34.2)	
*Baseline data*					
The study patients characteristics are described including baseline PRO scores	No	23 (35.4)	14 (25.5)	37 (30.8)	.241
	Yes	42 (64.6)	41 (74.5)	83 (69.2)	
*Outcomes and estimation*					
Are PRO outcomes also reported in a graphical format?	No	26 (40)	15 (27.3)	41 (34.2)	.143
	Yes	39 (60)	40 (72.7)	79 (65.8)	
(Additional standards only for PRO as primary outcome) The analysis of PRO data accounts for survival differences between treatment groups if relevant^a^	No	1 (3.8)	1 (6.2)	2 (4.8)	.002
	Yes	0 (0)	7 (43.8)	7 (16.6)	
	N/A (if not relevant)	25 (96.2)	8 (50)	33 (78.6)	
(Additional standards only for PRO as primary outcome) Results are reported for all PRO domains(if multidimensional)and items identified by the reference instrument^a^	No	3 (11.5)	7 (43.7)	10 (23.8)	.031
	Yes	23 (88.5)	9 (56.3)	32 (76.2)	
(Additional standards only for PRO as primary outcome) The proportion of patients achieving predefined responder definitions is provided where relevant^a^	No	1 (3.9)	3 (18.7)	4 (9.5)	.198
	Yes	7 (26.9)	2 (12.5)	9 (21.4)	
	N/A (if not relevant)	18 (69.2)	11 (68.8)	29 (69.1)	
**Discussion**					
*Limitations*					
The limitations of the PRO components of the trial are explicitly discussed	No	42 (64.6)	33 (60)	75 (62.5)	.603
	Yes	23 (35.4)	22 (40)	45 (37.5)	
*Generalizability*					
Generalizability issues uniquely related to the PRO results are discussed	No	28 (43.1)	26 (47.3)	54 (45)	.645
	Yes	37 (56.9)	29 (52.7)	66 (55)	
*Interpretation*					
Are PRO interpreted?(Not only restated)	No	19 (29.2)	12 (21.8)	31 (25.8)	.355
	Yes	46 (70.8)	43 (78.2)	89 (74.2)	
The clinical significance of the PRO findings is discussed	No	44 (67.7)	35 (63.6)	79 (65.8)	.641
	Yes	21 (32.3)	20 (36.4)	41 (34.2)	
Methodology used to assess clinical significance	Anchor based (eg minimal important difference)	15 (23.1)	13 (23.6)	28 (23.3)	.251
	Distribution based (e.g effect size)	6 (9.2)	3 (5.5)	9 (7.5)	
	Both	0 (0)	3 (5.5)	3 (2.5)	
	Other	0 (0)	1 (1.8)	1 (0.8)	
	Missing	44 (67.7)	35 (63.6)	79 (65.9)	
The PRO results is discussed in the context of the other clinical trial outcomes	No	4 (6.1)	15 (27.3)	19 (15.8)	.002
	Yes	61 (93.9)	40 (72.7)	101 (84.2)	
**Other information**					
*Protocol*					
(Additional standards only for PRO as primary outcome) A copy of the instrument is included if it has not been published previously (It could be found in the article appendix or in the online version of the paper)^a^	No	13 (50)	2 (12.5)	15 (35.7)	<.001
	Yes	13 (50)	0 (0)	13 (31)	
	N/A (if the instrument is already published or known in the literature)	0 (0)	14 (87.5)	14 (33.3)	

^a^ Percentage for these items was calculated by considering only the RCTs with PRO as primary endpoint (n = 42), that is, 26 RCTs published between January 2004 and March 2012 and 16 RCTs published from April 2012 to February 2019.

Evaluating the level of PRO reporting according to the CONSORT‐PRO extension, we observed, for all of the items, an improvement in the studies published after the publication of the CONSORT‐PRO extension, compared with those published before. Major differences were observed in two key items: the statistical methods for dealing with missing data were reported in 36.6% of newer RCTs as compared to 21.5% of older RCTs and PRO‐specific limitations were discussed in 46.3% vs 32.9% of RCTs, respectively (Table 5).

**TABLE 5:** Level of PRO reporting by year (2013) of publication of the CONSORT‐PRO extension

CONSORT‐PRO Extension Item	RCTs before CONSORT‐PRO publication (2004‐2013) (n = 79) No (%)	RCTs after CONSORT‐PRO publication (2014‐2019) (n = 41) No (%)
P1b. The PRO should be identified in the abstract as a primary or secondary outcome.	72 (91.1)	38 (92.7)
P2b. The PRO hypothesis should be stated, and relevant domains should be identified if applicable.^a^	25 (31.7)	14 (34.2)
P6a. Evidence of PRO instrument validity and reliability should be provided or cited if available.^b^	55 (69.6)	32 (78.0)
P6aa. This is the mode of administration, including the person completing the PRO and the methods of data collection (paper, telephone, electronic, and other).^b^, ^c^	15 (19.0)	9 (22.0)
P12a. Statistical approaches for dealing with missing data are explicitly stated.	17 (21.5)	15 (36.6)
P20/21. PRO‐specific limitations and implications for generalizability and clinical practice should be discussed.	26 (32.9)	19 (46.3)

Abbreviations: CONSORT, Consolidated Standards of Reporting Trials; PRO, patient‐reported outcome; RCT, randomized controlled trial.

^a^ This percentage was calculated on the basis of all studies (including those explicitly reporting an exploratory evaluation, for which this item would be rated as not applicable).

^b^ Items P6a and P6aa were combined in the original CONSORT‐PRO extension; however, for the purposes of this study, to better appraise the proportion of RCTs providing evidence on the validity of the PRO instrument but not further describing how this was administered to patients, this item was split into two items.

^c^ In case of studies using multiple PRO measures, we evaluated this as “yes” if at least one measure was validated.

## 4 DISCUSSION

Between April 2012 and February 2019, a total of 55 PCa RCTs have been published using PRO, of which nearly half (43.6%) can be considered as high‐quality RCTs for use of PROs. Of these 24 trials, only 13 (54.2%) have been reported in the most recent EAU Guidelines for PCa. The majority of RCTs were conducted in the advanced and metastatic PCa setting. Six RCTs were specifically conducted in men with localized PCa. Overall QoL and erectile, urinary, and bowel function were the most commonly reported PRO. The FACT‐P, EPIC‐26, and EORTC QLQ‐C30 and/or QLQ‐PR25 were the most commonly used measurement tools. An overall improvement in the reporting of the evidence for PROs was noted.

As highlighted by Kluetz et al cancer clinical trials have mainly focused on overall survival and measures of tumor growth or reduction to assess the efficacy of a specific treatment.^14^ However, the balance between improving disease symptoms and introducing symptomatic toxicity affects how patients function in their daily lives and hence affects their HRQoL. Kluetz and colleagues suggest to focus on three distinct measures of well‐defined concepts: symptomatic adverse events, physical function, and disease‐related symptoms. As shown in Table 2, these outcomes are measured in several of the included PCa RCTs, however, only the ACTRN12611000^15^ and the SPARTAN^16^ trials specifically reported on all three outcomes.

It is important to note that the majority of trials used validated tools, with FACT‐P, EPIC‐26, and EORTC QLQ‐C30 being the most commonly used. Those studies that were included in the EAU guidelines had all used at least one of these three measurement tools. However, these validated HRQoL questionnaires were less common in trials of men with localized PCa. The latter focused more on specific adverse events such as erectile or bowel function. Nevertheless, the CHHiP trial measured general HRQoL with FACT‐P^17^ and the TROG 03.04 RADAR trial measured QoL with EORTC QLQ‐C30 and its PCa module.^18^

There seems to be a need for PRO‐specific guidance for the different disease‐specific stages of PCa. There are emerging international standards^19^ to help generate robust data with more focus on patient engagement and the EAU is already undertaking work to develop core outcome sets for PCa, including both clinician‐reported outcomes and PROs.^4^, ^5^, ^20^ With improved methodology and practice, and increasing patient engagement, high quality and clinically meaningful generation of PRO data will become the norm for PCa clinical studies^21^ and help increase their clinical impact in every disease stage. This will ensure that more high‐quality PRO studies will also be incorporated in the recommendations of guidelines offices, which also uses robust evidence assessment methods. A modified Grading of Recommendations Assessment, Development and Evaluation (GRADE) is currently used by the EAU. This approach allows for a transparent assessment of how recommendation statements have been developed, whereby overall quality of the evidence along with magnitude of effect, certainty of the results, balance between desirable and undesirable outcomes, impact of patient values, and preferences on the intervention, and certainty of those patient values and preferences result in guideline recommendations.^7^ Inclusion of PRO studies into the guidelines thus provides a reflection on the quality of the evidence and the reporting of PROs.

It is important to note that a patient‐centred treatment recommendation should consider both clinical (ie survival) and HRQoL outcomes. Considering these HRQoL aspects relies on understanding patients’ values, needs, and experience of the disease and integrate them to formulate an optimal treatment strategy. Quality of life remains subjective and validated PROMs are of critical importance to guide these tailored interventions to improve patients’ well‐being. The rising importance of PROs is already captured for localized disease by a systematic review, coordinated by the EAU PCa Guidelines panel, evaluating the effect of primary treatment on HRQoL.^22^ The same PROMs as reported in our systematic review were captured, emphasizing their importance and systematic use. Such evaluation and recommendations are lacking for advanced and metastatic disease, where HRQoL becomes even more important. Methodology for reporting of PRO and HRQoL for these different disease stages does not need to differ, but there is a clear need for a broader consensus on its use in all disease stages so that it can be integrated with the existing clinical outcomes.

In addition to RCT data, it is of interest to note that there is an increased interest in using real world data to support regulatory decision‐making—including the EAU guidelines. Information about how a patient feels and functions, as captured directly from patients themselves, is however also often missing in real world data (eg observational data or hospital data). For example, PROs were only collected in 14% of recent postauthorisation safety studies.^23^ Harnessing the patient voice through the use PROs in “big data” indeed implies the need to align PRO measurements (PROMs) between RCTs and real world data.^24^

With respect to the methodological assessment of PRO use in PCa RCTs, data from this study suggests that completeness of PRO reporting has improved over the last years, as documented by the higher proportion of high‐quality RCTs published from 2012 onward. Indeed, while only 20% of PCa RCTs published between 2004 and 2012 were considered as high quality, this percentage has more than doubled for the more recent RCTs. This may be partly explained by the publication of the ISOQOL recommended standards^10^ and the subsequent CONSORT‐PRO criteria^11^ in 2013, which may have guided and helped investigators to increase the completeness of PRO reporting. Indeed, journal endorsement and author use of CONSORT‐PRO extension has been demonstrated to be associated with improved PRO reporting.^25^

This study has limitations. First, despite our comprehensive search strategy, it is possible that some RCTs with a PRO component might have been missed. Another limitation is the exclusion of non‐English language published papers. However, it is unlikely that such omission would have significantly altered the conclusion of this review.^26^ A strength of the current review is that we used a formal, replicable approach to evaluate PRO reporting of PCa RCTs. Since all studies use different reporting criteria and methods, the information was extracted and assessed by two independent researchers. In case of inconsistencies, a third arbiter helped achieving consensus. Also, by using state of the art and well‐established international recommendations for PRO reporting, we were able to identify the proportion of studies that are most likely to robustly inform patient care.

## 5 CONCLUSION

We observed an important improvement in the reporting of the evidence for PROs during the last 7 years, of which only a small proportion of high‐quality PRO trials made it into the EAU PCa Guidelines. The most commonly used measurements focused on overall HRQoL and were predominantly used in RCTs of men with advanced PCa, whereas for RCTs in men with localized PCa the focus was more on adverse effects. Given the increasing recognition that the patients’ voice in clinical research needs to be heard, there is a need for better guidance as to how to include and measure PRO in “big data” and guidelines—an answer which may be delivered by the EAU‐led PIONEER Consortium.

## CONFLICT OF INTEREST

None to be declared.

## AUTHOR CONTRIBUTIONS

Data collection: FS, LM, KB; data analysis: Fs, FC, FE, MVH; manuscript drafting: MVH, LM, KB; final approval of manuscript: MVH, FS, LM, KB, FC, MS, FE.

## Supporting information

Supplementary Material

## Data Availability

All data were taken from published manuscripts and can be obtained upon request by contacting f.sparano@gimema.it.
