# Supplementary material for: Harnessing the patient voice in prostate cancer research: Systematic review on the use of patient‐reported outcomes in randomized controlled trials to support clinical decision‐making
Source: Cancer Med. 2020 Apr 25;9(12):4039–58. doi: 10.1002/cam4.3018 (PMC7300413; doi:10.1002/cam4.3018)
Supplement: Supplementary file 1 — Supplementary Material [file CAM4-9-4039-s001.PDF]

# PROMOTION Registry

## (Patient-Reported Outcome Measurements Over Time In ONcology)

This Project is supported by the **GIMEMA**

**Study website**: http://promotion.gimema.it/

# Data Extraction Form

The Data extraction Form must be completed in every part.

Please, complete the Form using the information available in the relevant paper/s

The present document is a working hard copy of the web version of the "electronic-Data Extraction Form(**eDEF**)" that you will find in the online data collection system of the PROMOTION study website: http://promotion.gimema.it/

If you are an authorized Investigator of the PROMOTION Registry, please contact the PROMOTION data management team (E-mail: promotion@gimema.it) to receive your personal ID and password.

**IMPORTANT NOTE:**
An additional explanatory document, further clarifying criteria to rate items reported in this **eDEF**, is available from the PROMOTION data management team: (E-mail: promotion@gimema.it)

The "Data Extraction Form" is divided in three sections:

- **Section A** includes general information and descriptive characteristics of the study.
- **Section B** is based on the *International Society for Quality of Life Research* (ISOQOL) Consensus based standards for reporting PRO in RCTs (Brundage M, et al, Qual Life Res, 2013; 22: 1161-1175). These criteria are also the basis of the CONSORT PRO guidelines (Calvert M, et al, JAMA 2013; 309: 814-822).
- **Section C** is the *Cochrane Collaboration's Tool for Assessing Risk of Bias*. (Higgins JP, et al, BMJ 2011; 343: d5928)

**Journal:**______________________________

**Name of first author:**_________________

# Section A

## 1) Name of Cooperative Group/s

(If available in the paper, the name of the group/s on behalf of which the RCT was conducted should be reported).

- ☐ EORTC
- ☐ SWOG
- ☐ CALGB/ALLIANCE
- ☐ NCI
- ☐ NCI-C
- ☐ MRC-UK
- ☐ ECOG
- ☐ other, please specify: ___________________________________________________________________

## 2) Study Location

**2.1)** Main country: _________________________________________________________________________

**2.2)** International (If more than one country).

- ○ yes
- ○ no

## 3) Industry supported (fully or in part)

(Defined as "yes" if one of the authors has an affiliation with a pharma company or there is an acknowledgment to a pharma company in the paper).

- ○ yes
- ○ no

## 4) Primary endpoint/s

- ☐ objective response rate
- ☐ overall survival
- ☐ progression free-survival
- ☐ disease free survival
- ☐ time to treatment failure (including disease progression, treatment toxicity, death)
- ☐ PRO (including QOL or symptoms relief)
- ☐ other, please specify: ____________________________________________________________________

## 5) Difference between treatment arms in the primary endpoint

We applied 'Yes' if a trial showed at least a statistically significant difference in the primary endpoint (whatever it is).

- ○ yes
- ○ no

**5.1)** Any comment?

(If none, report "none")

_________________________________________________________________________________________

## 6) Overall Survival (OS) difference favoring experimental treatment

- ○ yes
- ○ no
- ○ N/A (e.g., in case OS was not an endpoint)

**7) Summary of <u>main clinical</u> results**
(Only describe statistically significant outcomes other than PRO; i.e. OS, PFS. If none report "none").

______________________________________________

______________________________________________

**8) Age of Patients**
(Report available data on patients'age).

____________

**9) Gender of patients**

**9.1) Males n: (absolute total number)**
(Report "0" if not available).

____________

**9.2) Females n: (absolute total number)**
(Report "0" if not available).

____________

**9.3) Any comment?**
(If none, report "none").

______________________________________________

______________________________________________

**10) Broad Treatment type**

- ☐ radiotherapy
- ☐ surgery
- ☐ chemotherapy
- ☐ targeted therapy
- ☐ hormonal therapy
- ☐ other, please specify: ______________________________

**11) Specific Treatments being compared between treatments arms**
(Report details such as name of drugs, doses, duration of treatment).

______________________________________________

______________________________________________

**12) Disease stage**
(Defined as metastatic if distant metastases were present).

- ☐ metastatic/advanced
- ☐ non-metastatic/local
- ☐ both
- ☐ unclear

**13) Overall Trial Sample size**
(Overall number of patients recruited in the study regardless of those with a baseline PRO assessment).

______________________________________________

**14) PRO sample size**
(Actual number of patients with a baseline PRO evaluation).

______________________________________________

**15) PRO endpoint**

- ○ primary
- ○ secondary

**16) Secondary paper on PRO**

(Defined as "yes" if there is a secondary paper focused exclusively on PRO).

- ○ yes
- ○ no

**17) Type of PRO instrument/s used**

- ☐ EORTC instruments
- ☐ FACT instruments
- ☐ VAS
- ☐ MDASI
- ☐ others

**18) Specific names of all PRO instruments used**

__________________________________________________________________

__________________________________________________________________

__________________________________________________________________

**19) Summary of <u>PRO</u> results**

(Briefly describe the main PRO findings. Only statistically significant or clinically significant results should be discussed. If none report "none").

__________________________________________________________________

__________________________________________________________________

__________________________________________________________________

**20) PRO difference between treatment arms?**

(We applied "Yes" if a trial showed at least a statistically significant difference in one PRO domain at any time point assessment throughout study period).

- ○ yes, broadly favoring experimental treatment/s
- ○ yes, broadly favoring standard treatment/s
- ○ no differences at all
- ○ N/A (e.g., in case no findings were reported or the interpretation is unclear)

**21) If statistically significant PRO difference exists, in which domain?**

- ○ only symptoms (e.g. either single or multiple symptoms scales)
- ○ only domains other than symptoms (e.g. physical or emotional functioning or QOL)
- ○ both domains (symptoms + domains other than symptoms)
- ○ no differences at all
- ○ N/A (e.g., in case no findings were reported)

**22) Length of PRO assessment during RCT**

- ○ up to 6 months
- ○ up to 1 year
- ○ more than 1 year
- ○ unknown

# Section B

| Reporting Standard Category | **For all studies**<br>**(Regardless of whether the PRO is a $1^{o}$ or $2^{o}$ outcome)** | **Additional standards only for PRO as primary outcome** |
|---|---|---|
| **Title & abstract** | The PRO should be identified as an outcome in the abstract.<br><br>☐ Yes<br>☐ No | The title of the paper should be explicit as to the RCT including a PRO.<br><br>☐ Yes<br>☐ No |
| **Introduction, background and objectives** | The PRO hypothesis should be stated and should specify the relevant PRO domain(s) if applicable.<br><br>☐ Yes<br>☐ No<br>☐ N/A (if explorative) | The introduction should contain a summary of PRO research that is relevant to the RCT.<br><br>☐ Yes<br>☐ No<br><br>Additional details regarding the hypothesis should be provided, including the rationale for the selected domain(s), the expected direction(s) of change, and the time points for assessment.<br><br>☐ Yes<br>☐ No |
| ***Methods*** | | |
| **Outcomes** | The mode of administration of the PRO tool and the methods of collecting data (e.g. telephone, other) should be described.<br><br>☐ Yes<br>☐ No<br><br>Electronic mode of PRO administration<br><br>☐ Yes<br>☐ No<br>☐ N/A<br><br>The rationale for choice of the PRO instrument used should be provided.<br><br>☐ Yes<br>☐ No<br><br>Evidence of PRO instrument validity and reliability should be provided or cited.<br><br>☐ Yes, for all PRO instruments<br>☐ No, only for some PRO instruments<br>☐ No<br><br>The intended PRO data collection schedule should be provided.<br><br>☐ Yes<br>☐ No<br><br>PROs should be identified in the trial protocol; post-hoc analyses should be identified.<br><br>☐ Yes<br>☐ No<br><br>The status of PRO as either a primary or secondary outcome should be stated.<br><br>☐ Yes<br>☐ No<br>☐ Unclear | A citation for the original development of the PRO instrument should be provided.<br><br>☐ Yes, for all PRO instruments<br>☐ No, only for some PRO instruments<br>☐ No<br><br>Windows for valid PRO responses should be specified and justified as being appropriate for the clinical context.<br><br>☐ Yes<br>☐ No |

| | | |
|---|---|---|
| **Sample size** | | There should be a power/sample size calculation relevant to the PRO based on a clinical rationale (e.g. anticipated effect size).<br><br>☐ Yes<br>☐ No |
| **Statistical methods** | There should be evidence of appropriate statistical analysis and tests of statistical significance for each PRO hypothesis tested.<br><br>☐ Yes<br>☐ No<br>☐ N/A (if PRO hypothesis were not stated)<br><br>For statistically significant PRO results, the magnitude of the effect size should be stated.<br><br>☐ Yes<br>☐ No<br>☐ N/A (for not statistically significant results)<br><br>Statistical approaches for dealing with missing data should be explicitly stated.<br><br>☐ Yes<br>☐ No<br><br>The extent of missing data should be stated.<br><br>☐ Yes<br>☐ No | The manner in which multiple comparisons have been addressed should be provided.<br><br>☐ Yes<br>☐ No |
| ***Results*** | | |
| **Participant flow** (a diagram is strongly recommended) | A flow diagram or a description of the allocation of participants and those lost to follow-up should be provided for PROs specifically.<br><br>☐ Yes<br>☐ No<br><br>The reasons for missing data should be explained.<br><br>☐ Yes<br>☐ No | |
| **Baseline data** | The study patients' characteristics should be described, including baseline PRO scores.<br><br>☐ Yes<br>☐ No | |
| **Outcomes and estimation** | Are PRO outcomes also reported in a graphical format?<br><br>☐ Yes<br>☐ No | The analysis of PRO data should account for survival differences between treatment groups if relevant.<br><br>☐ Yes<br>☐ No<br>☐ N/A (if not relevant)<br><br>Results should be reported for all PRO domains (if multi-dimensional) and items identified by the reference instrument (i.e. not just those that are statistically significant).<br><br>☐ Yes<br>☐ No |

| | | |
|---|---|---|
| | | The proportion of patients achieving pre-defined responder definitions should be provided where relevant.<br><br>☐ Yes<br>☐ No<br>☐ N/A (if not relevant) |
| ***Discussion*** | | |
| **Limitations**<br><br><br><br><br>**Generalizability**<br><br><br><br><br>**Interpretation** | The limitations of the PRO components of the trial should be explicitly discussed.<br><br>☐ Yes<br>☐ No<br><br>Generalizability issues uniquely related to the PRO results should be discussed.<br><br>☐ Yes<br>☐ No<br><br>Are the PRO interpreted? (not only re-stated)<br><br>☐ Yes<br>☐ No<br><br>The clinical significance of the PRO findings should be discussed<br><br>☐ Yes<br>☐ No<br><br>Methodology used to assess clinical significance.<br><br>☐ Anchor based<br>☐ Distribution based<br>☐ Both<br>☐ Other, please specify<br>______________________________<br>______________________________<br>______________________________<br>______________________________<br><br>The PRO results should be discussed in the context of the other clinical trial outcomes.<br><br>☐ Yes<br>☐ No | |
| **Other Information** | | |
| **Protocol** | | A copy of the instrument should be included if it has not been published previously (It could be found in the article appendix or in the online version of the paper).<br><br>☐ Yes<br>☐ No |

# Section C

## ASSESSMENT OF RISK OF BIAS

(Assessment should be made only for the MAIN clinical outcome of the study)

*Selection bias*

- ✓ **Random sequence generation**
  - o Low
  - o High
  - o Unclear
- ✓ **Allocation concealment**
  - o Low
  - o High
  - o Unclear

*Performance bias*

- ✓ **Blinding of participants and personnel**
  - o Low
  - o High
  - o Unclear

*Detection bias*

- ✓ **Blinding of outcome assessment**
  - o Low
  - o High
  - o Unclear

*Attrition bias*

- ✓ **Incomplete outcome data**
  - o Low
  - o High
  - o Unclear

*Reporting bias*

- ✓ **Selective reporting**
  - o Low
  - o High
  - o Unclear

*Other bias*

(Please specify other sources of bias not addressed. If none report "none")

______________________________________________

- o Low
- o High
- o Unclear
- o N/A
